# Supplementary material for: Bias‐Engineered Synthetic Antiferromagnets Hosting Sub‐20 nm Zero‐Field Skyrmions at Room Temperature
Source: Adv Sci (Weinh). 2026 May 27:e75825. Online ahead of print. doi: 10.1002/advs.75825 (PMC13335783; doi:10.1002/advs.75825)
Supplement: Supplementary file 1 — Supporting File 1: advs75825‐sup‐0001‐SuppMat.pdf. [file ADVS-9999-e75825-s002.pdf]

Supporting information for Bias-Engineered Synthetic Antiferromagnets Hosting sub-20 nm Zero-Field Skyrmions at Room Temperature

Emily Darwin Riccardo Tomasello Reshma Peremadathil Pradeep Mario Carpentieri Giovanni Finocchio Hans J. Hug**

E. Darwin, R. Peremadathil Pradeep, H. J. Hug
Empa, Swiss Federal Laboratories for Materials Science and Technology, Ueberlandstrasse 129, 8600 Dübendorf, Switzerland
Email Address: emily.darwin@empa.ch
Email Address: hans-josef.hug@empa.ch
R. Tomasello, M. Carpentieri
Department of Electrical and Information Engineering, Politecnico di Bari, I-70125 Bari, Italy
G. Finocchio
Department of Mathematical and Computer Sciences, Physical Sciences and Earth Sciences, University of Messina, I-98166 Messina, Italy
R. Peremadathil Pradeep, H. J. Hug
Department of Physics, University of Basel, Klingelbergstrasse 82, 4056 Basel, Switzerland

1 Magnetic Anisotropy and Stacking-Order Effects for Ru/Co/Pt Multilayers

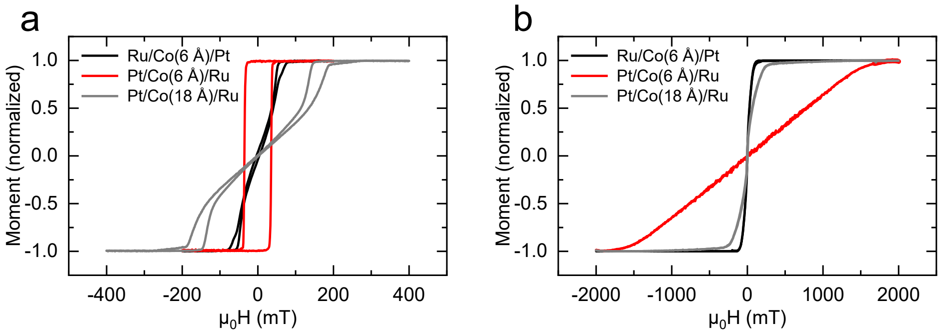

Figure S1: Hysteresis loops taken via out-of-plane (a) and in-plane (b) VSM showing data for $[Ru(13)/Co(6)/Pt(6)]_{\times 6}$ (black curve), $[Pt(6)/Co(6)/Ru(13)]_{\times 6}$ (red curve), and $[Pt(6)/Co(18)/Ru(13)]_{\times 6}$ (gray curve).

Figure S1a displays the out-of-plane (easy-axis) and Figure S1b the in-plane (hard-axis) hysteresis loops measured by vibrating sample magnetometry (VSM) for three different six-repetition multilayers. The black curve corresponds to the $[Ru(13)/Co(6)/Pt(6)]_{\times 6}$ multilayer studied in this work, specifically the ferromagnetic (FM) multilayer (ML). The red curve corresponds to the system stacked in reverse order, with Pt below and Ru above the Co, $[Pt(6)/Co(6)/Ru(13)]_{\times 6}$, similar to the synthetic antiferromagnet (SAF) bias system. The gray curve represents a ML also with Pt below and Ru above the Co layer, however, with an increased Co thickness: $[Pt(6)/Co(18)/Ru(13)]_{\times 6}$. From the VSM loops,

we extract a uniaxial anisotropy constant of $K_{\mathrm{u}}^{\mathrm{Pt/Co/Ru}} = 1.26$ MJ/m^3 for the reversed-order multilayer, which is substantially larger than the anisotropy of the Ru/Co/Pt system investigated here, $K_{\mathrm{u}}^{\mathrm{Ru/Co/Pt}} = 0.68$ MJ/m^3. We attribute this enhanced anisotropy in the Pt/Co/Ru stack to improved layer growth when Pt forms the seed interface.

Consistent with the higher anisotropy, we found that the reversed-order Pt/Co/Ru multilayer does not host skyrmions for a Co layer thickness of 6 Å. Only when the Co thickness is increased to 18 Å do skyrmions appear, at an applied out-of-plane field of around 150 mT. This demonstrates the strong sensitivity of skyrmion stability to the interfacial anisotropy set by the ML stacking order.

2 Bias Layer Stability Considerations

2.1 Skyrmion Formation Energy Densities

To obtain skyrmions in our Ru/Co/Pt FM ML with a saturation magnetization $M_{\mathrm{s}} = 920$ kA/m (see Table 1 in the main manuscript), an external field of $\mu_0 H_{\mathrm{sk}} \approx 50$ mT must be applied (see the hysteresis loop in Figure 2a of the main manuscript). This corresponds to an energy density

$$\epsilon_{\mathrm{sk}} = \frac{1}{2}\mu_0 H_{\mathrm{sk}} M_{\mathrm{s}} = 23\ \mathrm{kJ/m}^3, \tag{1}$$

which must be supplied by the stabilizing field.

If the same energy were provided by an interfacial exchange field, e.g., by a suitable biasing layer, then the corresponding *interfacial* energy density would be

$$\epsilon_{\mathrm{sk}} t_{\mathrm{Co,tot}} = 23\ \mathrm{kJ/m}^3 \cdot 36\ \text{Å} = 8.28 \times 10^{-5}\ \mathrm{J/m}^2, \tag{2}$$

where $t_{\mathrm{Co,tot}} = 36$ Å is the total Co thickness of the FM ML. In our system, this interfacial exchange field is provided by the antiferromagnetically coupled SAF bias system via the RKKY interaction through a 6 Å Ru spacer layer.

For the biasing concept to work, the SAF bias system must remain in a *uniform* magnetization state. If it were to break up into maze domains, it would no longer generate a uniform exchange field acting on the FM ML, even though the antiferromagnetic interlayer exchange would remain intact within the SAF bias system. Such a domain state could occur if the zero-field domain pattern of the FM ML (Figure 2b of the main manuscript) were imprinted into the SAF bias system. In that case, zero-field skyrmions could not be stabilized, and the biasing concept would fail.

2.2 Stability of the SAF Bias System Against Domain Imprinting

To evaluate the robustness of the SAF bias system, we estimate the energy required to generate domains in the SAF itself. The domain wall energy density of the SAF bias system is

$$\sigma_{\mathrm{dw}} = 4\sqrt{A K_{\mathrm{eff}}} = 6.92 \times 10^{-3}\ \mathrm{J/m}^2, \tag{3}$$

where an exchange stiffness $A = 8$ pJ/m was assumed and the experimentally obtained effective anisotropy of $K_{\mathrm{eff}} = 374$ kJ/m^3 was used. This value was calculated from the in-plane VSM hysteresis loop of the SAF bias system in Figure S2. Assuming linear stripe domains in the FM ML with a domain width of 133 nm (the domain width was obtained from an FFT analysis of the maze domain pattern shown in Figure 2b of the main manuscript), the number of domain walls in 1 m film surface length is

$$N_{\mathrm{dw}} = \frac{1\,\mathrm{m}}{133\ \mathrm{nm}} = 7.52 \times 10^6.$$

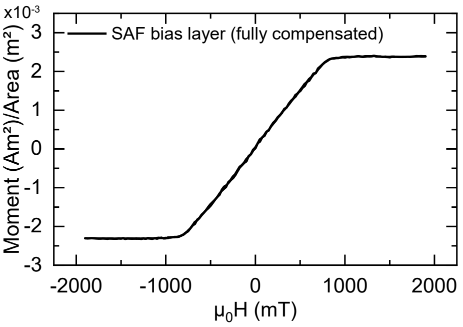

Figure S2: In-plane VSM hysteresis loop, used to work out the anisotropy of the fully compensated SAF bias system (shown in Figures 1a and b of the main manuscript).

Therefore considering a $1\,\mathrm{m}^2$ surface area of the film, we obtain the total wall area through the film thickness as

$$A_{\mathrm{dw}} = N_{\mathrm{dw}} \cdot 1\,\mathrm{m} \cdot 2.2\ \mathrm{nm} = 1.65 \times 10^{-2}\ \mathrm{m}^2,$$

per m^2 film surface area, where 2.2 nm is the total thickness of both Co layers of the partially compensated SAF bias system.

Multiplying by σ_{dw} gives a total domain wall energy of

$$E_{\mathrm{SAF}}^{\mathrm{imprint}} = 1.15 \times 10^{-4}\ \mathrm{J/m}^2, \quad (4)$$

per surface area, for the domain pattern imprinted into the biasing SAF. Note that here we neglect the additional magnetostatic energy arising from opposite magnetic moments in the Co sub-layers of the SAF bias system.

By contrast, the interfacial energy needed to stabilize skyrmions in the FM ML is $\epsilon_{\mathrm{sk}} t_{\mathrm{Co,tot}} = 0.83 \times 10^{-4}\ \mathrm{J/m}^2$ (Equation 2), which is larger than the energy required to form domains in the SAF bias system. Thus, the SAF remains in a homogeneous state, validating the bias-layer approach for the Ru/Co/Pt multilayer.

2.3 Implications for Reversed-Order Multilayers

The reversed-order stack with the same Co thickness of 6 Å exhibits an effective anisotropy of 728 $\mathrm{kJ/m}^3$, as extracted from the hard-axis magnetization loop shown in Figure S1b (red curve). This comparatively large anisotropy yields a conventional square, easy-axis magnetization loop with 100% remanence and does not support skyrmion formation (red curve in Figure S1a). Skyrmions can be stabilized in the reversed stacking sequence only when the Co layer thickness is increased to 18 Å. Under these conditions, the magnetization loop displays the characteristic signatures of a skyrmion-hosting multilayer, with skyrmions appearing at fields of approximately 150 mT. However, this comes at the cost of significantly more demanding biasing conditions. Both the required skyrmion-stabilizing field and the total Co thickness are substantially larger than those of the Ru/Co/Pt multilayer discussed in the main manuscript. Consequently, the energy per unit area increases to $7.47 \times 10^{-4}\ \mathrm{J/m}^2$, which is nine times higher than that of the Ru/Co/Pt stack order (Equation 2), and roughly twice the energy needed to imprint domains into the SAF. As a result, the SAF bias layer would no longer remain uniformly magnetized but would instead break up into domains, preventing it from providing a homogeneous RKKY exchange field. This analysis justifies our choice of the Ru/Co/Pt stacking order for enabling reliable SAF biasing and zero-field skyrmion stabilization.

3 Relevance of Background Subtraction for MFM Data Interpretation

The sensitivity of our MFM, operated with soft, high-quality-factor cantilevers under vacuum conditions, is enhanced by about two orders of magnitude compared with conventional ambient-condition MFM.[1] This exceptionally high sensitivity is crucial for detecting spin textures that generate only extremely weak stray fields, such as the SAF skyrmions investigated here.

However, MFM always measures the *sum* of all forces acting on the tip. In addition to the magnetic tip–sample interaction, the signal contains contributions from variations in van der Waals forces arising from sample topography when scanning at a constant average height. Furthermore, spatial variations in the magnetic moment that are unrelated to the spin texture under study, for example due to local magnetic layer thickness fluctuations or local tilts of the anisotropy axis, also generate stray field variations.[1] These non-idealities can easily mask the weak stray field of SAF skyrmions, whose magnetic contrast is strongly reduced due to the nearly compensated multilayer structure.

To reliably isolate the stray field signature of SAF skyrmions, a background subtraction was applied to all MFM datasets used in the main manuscript (except for the SAF ML alone, as at zero field, it was already in a saturated state). Figure S3 illustrates the effect of this procedure for the SAF skyrmions, where panel c corresponds to the background-subtracted data presented in Figure 5b of the main manuscript and subsequently used for quantitative analysis.

In summary, the combination of highly sensitive vacuum MFM, rigorous background subtraction, and quantitative MFM analysis is essential for unambiguously identifying SAF skyrmions and determining their dimensions.

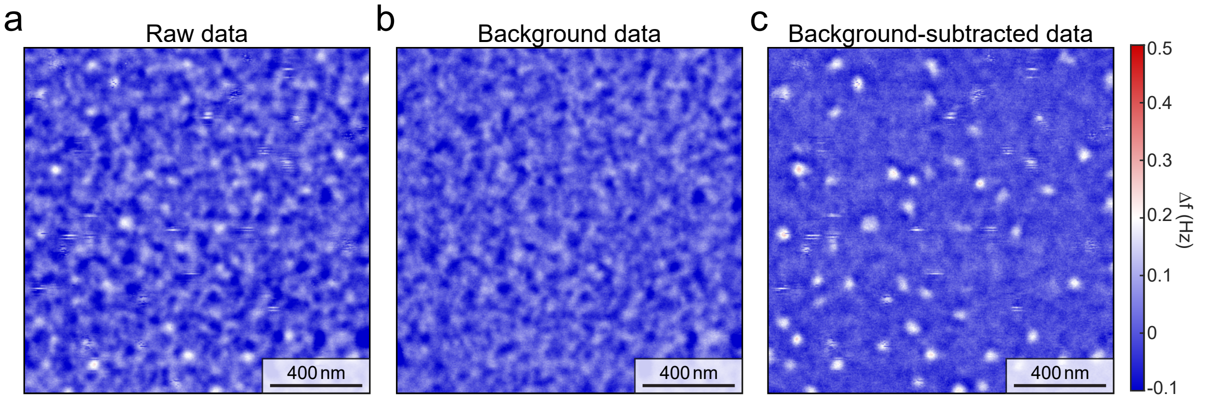

Figure S3: a Raw data of the SAF bias system + SAF ML taken at 0 mT via in-vacuum MFM. b The background data of the same area at 30 mT when the skyrmions have disappeared. c Background-subtracted data of the same area, i.e., the data in b subtracted from the data in a.

4 Compensated vs. Partially Compensated SAF Bias Layer

Figure S4 presents two hysteresis loops measured using out-of-plane VSM. The black curve corresponds to the SAF ML described in the main manuscript, with a SAF bias system beneath it that is partially compensated. The red curve, by contrast, represents a SAF ML with a fully compensated SAF bias system. In this case, the Co thickness within the SAF ML is 8 Å, though the magnetic moment has been scaled for comparison. The total magnetic moment per unit area is slightly higher for the fully compensated bias

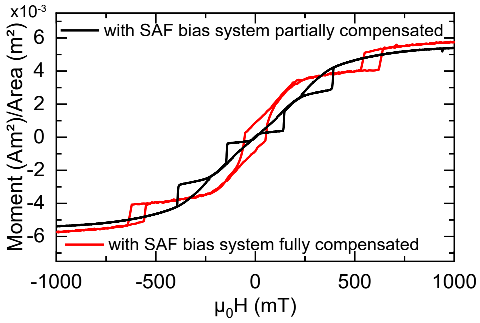

Figure S4: Hysteresis loops taken via out-of-plane VSM showing the SAF bias layer + SAF multilayer, with a fully compensated SAF bias layer (red curve) and a partially compensated SAF bias layer (black curve).

layer, owing to the additional 2 Å of Co in the lower sub-layer of the SAF bias system. Notably, in the fully compensated case, a finite remanence is observed at zero applied magnetic field, indicating that the whole system is not entirely compensated, likely due to interfacial effects between the two sub-systems. Conversely, when the compensation of the SAF bias system is intentionally broken by reducing the lower sub-layer thickness from 12 Å to 10 Å, the overall system becomes fully compensated, and no magnetic remanence is observed.

5 Skyrmion Core Polarity Control

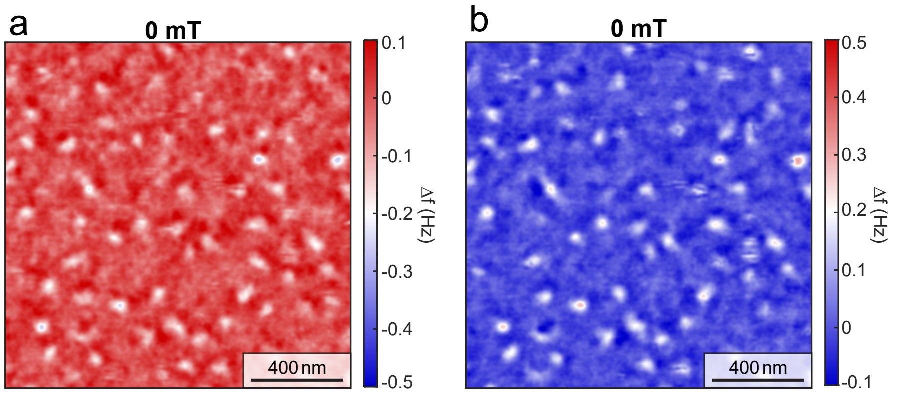

Figure S5: MFM data of the SAF bias system + SAF ML. a The sample was saturated in a north magnetic field, then measured at zero field. b The sample was saturated in a south magnetic field, then measured at zero field. Both measurements were performed with a south-oriented magnetic tip.

Figure S5 illustrates the controlled reversal of the skyrmion core orientation in the combined SAF bias system and SAF ML system. When the whole system is saturated in a north-directed magnetic field and subsequently returned to zero field, the thicker upper Co sub-layer of the SAF bias system is expected to retain a northward magnetization. Through the antiferromagnetic RKKY coupling, this sets the magnetization of the bottom Co trilayer of the SAF ML predominantly to the south, while the top Co trilayer aligns predominantly north. In this configuration, the skyrmions hosted by the SAF ML exhibit a north-oriented core in the bottom trilayer of the SAF ML and a south-oriented core in the top trilayer. This behavior is reflected in the MFM data shown in Figure S5a. The top trilayer appears predominantly red, corresponding to a

repulsive interaction with the south-magnetized MFM tip and therefore a local north-oriented magnetization. In contrast, the skyrmions appear blue, indicating an attractive interaction and thus a south-oriented core. In Figure S5b, the system was instead saturated in a south-directed magnetic field before returning to zero field. In this case, the remanent magnetization of the upper Co layer in the SAF bias system reverses, leading to the opposite orientation in all antiferromagnetically coupled layers. Consequently, the top Co trilayer in the SAF ML is predominantly blue, an attractive interaction with the south-magnetized tip, indicating a dominant southward magnetization. The skyrmions now appear red, corresponding to a repulsive force and therefore a north-oriented core. Together, the two datasets demonstrate that the skyrmion core orientation in the SAF ML can be fully and reversibly controlled by applying a saturating magnetic field in either the north or south direction.

References

[1] Y. Feng, P. M. Vaghefi, S. Vranjkovic, M. Penedo, P. Kappenberger, J. Schwenk, X. Zhao, A.-O. Mandru, H. Hug, *Journal of Magnetism and Magnetic Materials* **2022**, *551* 169073.
