# Supplementary figures and images for: Bias‐Engineered Synthetic Antiferromagnets Hosting Sub‐20 nm Zero‐Field Skyrmions at Room Temperature
Source: Adv Sci (Weinh). 2026 May 27:e75825. Online ahead of print. doi: 10.1002/advs.75825 (PMC13335783; doi:10.1002/advs.75825)

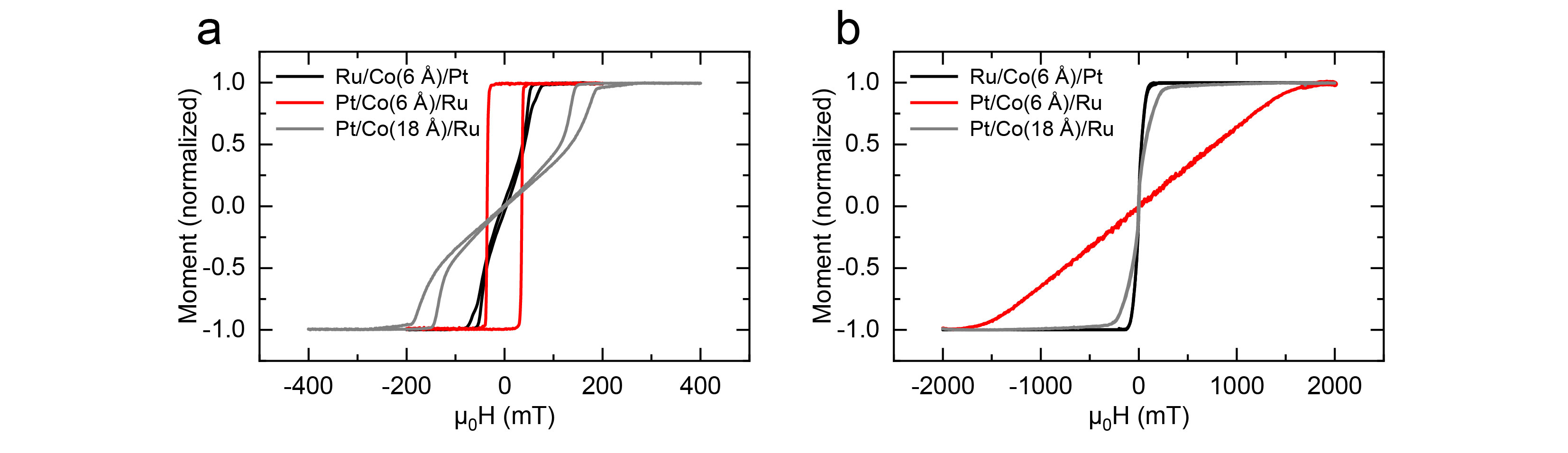

Supplement: Supplementary file 2 — Supporting File 2: advs75825‐sup‐0002‐FigureS1‐S5.zip. [file ADVS-9999-e75825-s001.zip › Supp_Figure1_1.jpg]

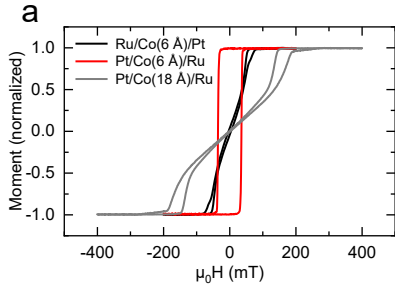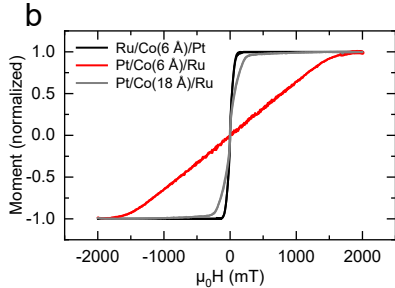

Supplement: Supplementary file 2 — Supporting File 2: advs75825‐sup‐0002‐FigureS1‐S5.zip. [file ADVS-9999-e75825-s001.zip › Supp_Figure1_1.pdf]

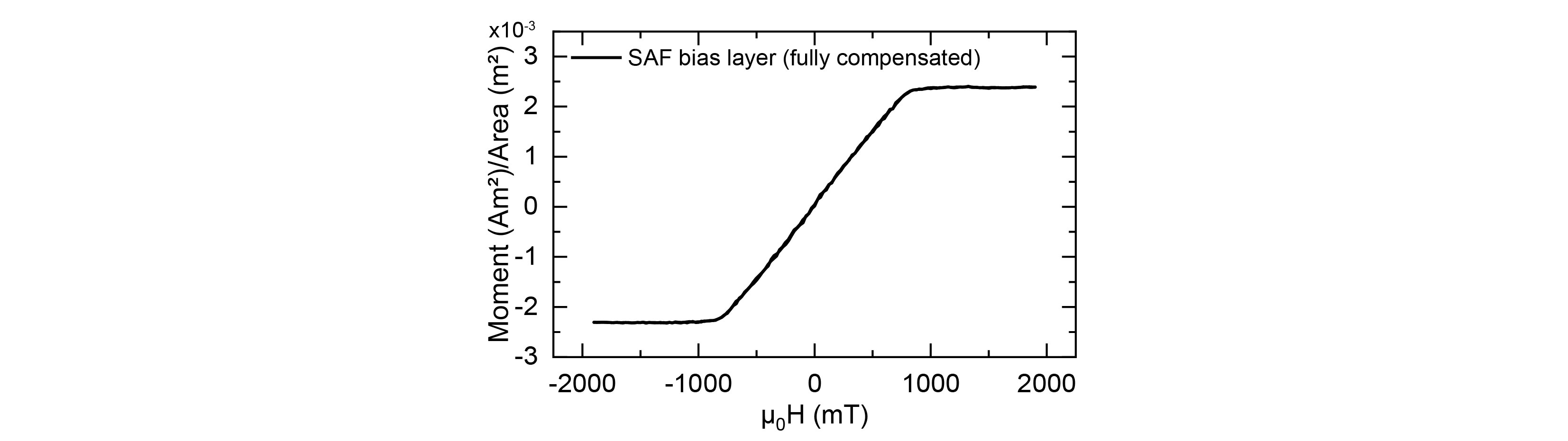

Supplement: Supplementary file 2 — Supporting File 2: advs75825‐sup‐0002‐FigureS1‐S5.zip. [file ADVS-9999-e75825-s001.zip › Supp_Figure2_1.jpg]

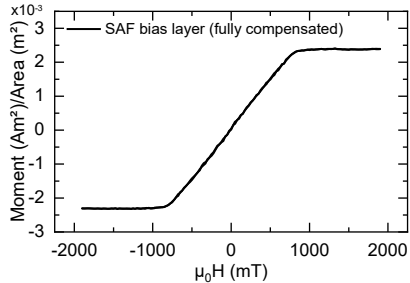

Supplement: Supplementary file 2 — Supporting File 2: advs75825‐sup‐0002‐FigureS1‐S5.zip. [file ADVS-9999-e75825-s001.zip › Supp_Figure2_1.pdf]

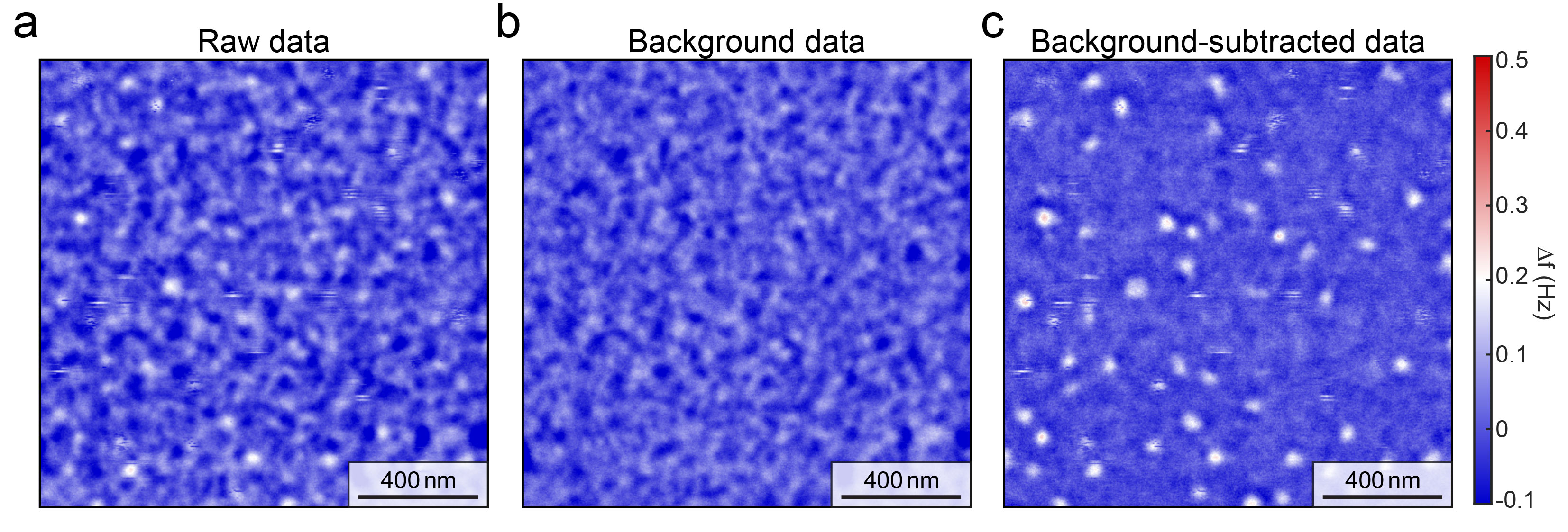

Supplement: Supplementary file 2 — Supporting File 2: advs75825‐sup‐0002‐FigureS1‐S5.zip. [file ADVS-9999-e75825-s001.zip › Supp_Figure3_1.jpg]

**a**

Raw data

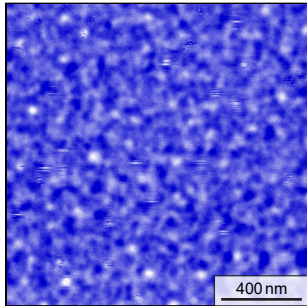**b**

Background data

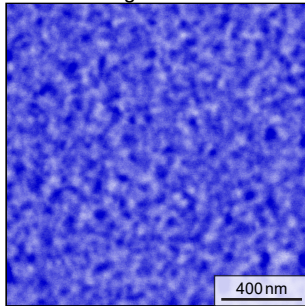**c**

Background-subtracted data

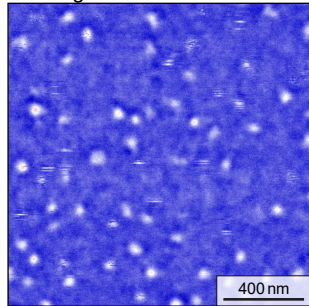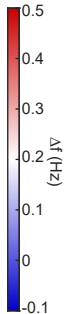

Supplement: Supplementary file 2 — Supporting File 2: advs75825‐sup‐0002‐FigureS1‐S5.zip. [file ADVS-9999-e75825-s001.zip › Supp_Figure3_1.pdf]

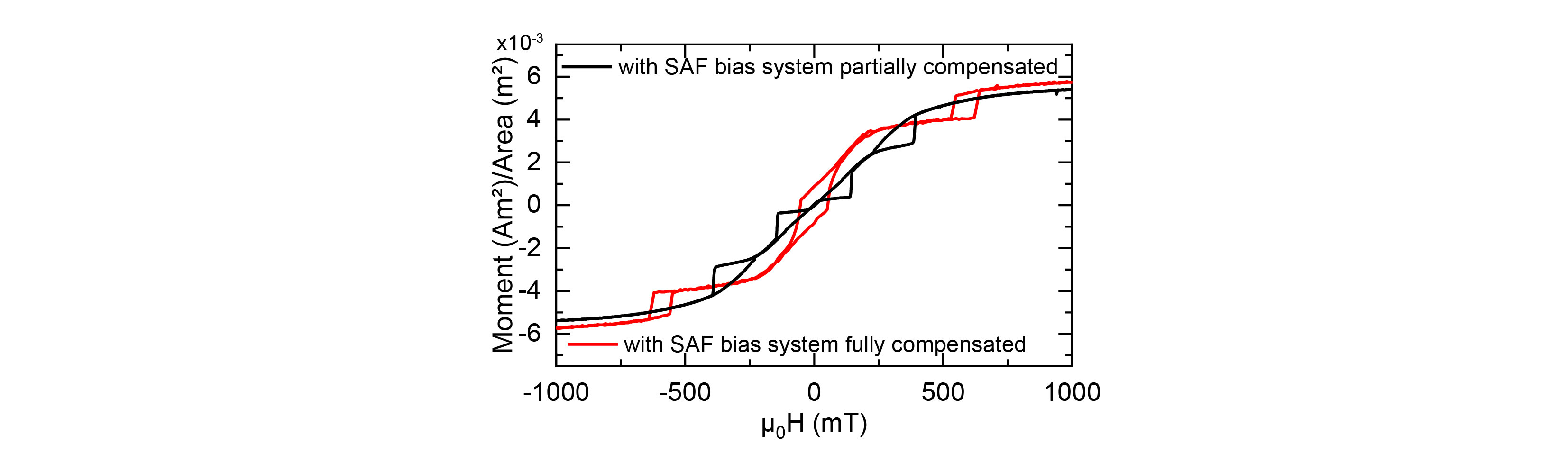

Supplement: Supplementary file 2 — Supporting File 2: advs75825‐sup‐0002‐FigureS1‐S5.zip. [file ADVS-9999-e75825-s001.zip › Supp_Figure4_1.jpg]

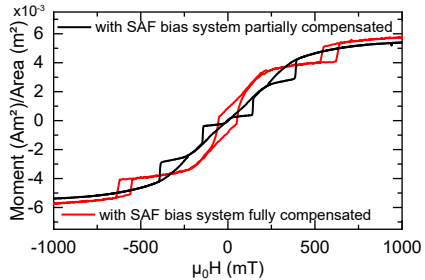

Supplement: Supplementary file 2 — Supporting File 2: advs75825‐sup‐0002‐FigureS1‐S5.zip. [file ADVS-9999-e75825-s001.zip › Supp_Figure4_1.pdf]

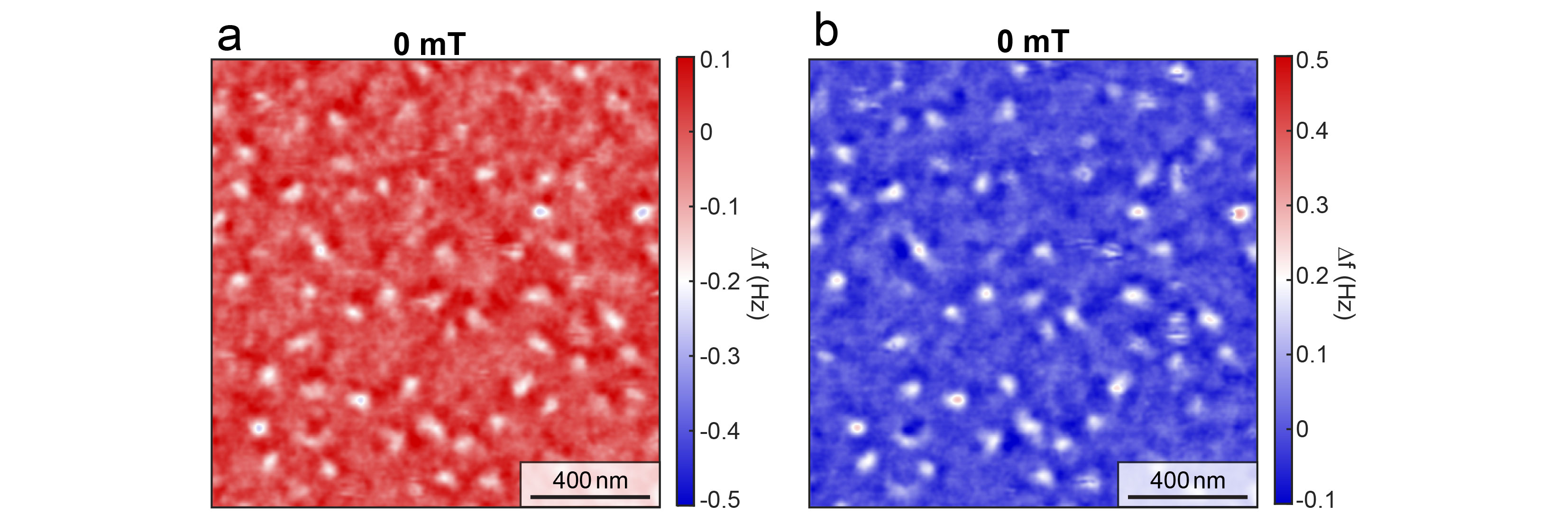

Supplement: Supplementary file 2 — Supporting File 2: advs75825‐sup‐0002‐FigureS1‐S5.zip. [file ADVS-9999-e75825-s001.zip › Supp_Figure5_1.jpg]

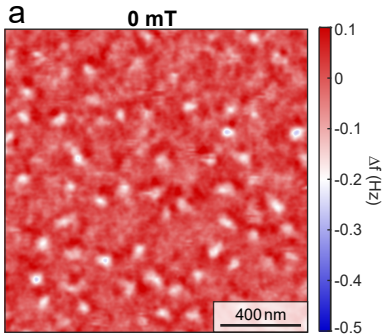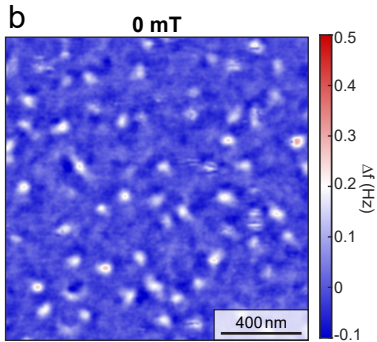

Supplement: Supplementary file 2 — Supporting File 2: advs75825‐sup‐0002‐FigureS1‐S5.zip. [file ADVS-9999-e75825-s001.zip › Supp_Figure5_1.pdf]
